# Effect of swearing on physical performance: a mini-review

**DOI:** 10.3389/fpsyg.2024.1445175

**Published:** 2024-11-11

**Authors:** Nicholas B. Washmuth, Richard Stephens, Christopher G. Ballmann

**Affiliations:** ^1^Department of Physical Therapy, Samford University, Birmingham, AL, United States; ^2^School of Psychology, Keele University, Keele, United Kingdom; ^3^Department of Human Studies, University of Alabama at Birmingham, Birmingham, AL, United States; ^4^Center for Engagement in Disability Health and Rehabilitation Sciences (CEDHARS), University of Alabama at Birmingham, Birmingham, AL, United States; ^5^University Center for Exercise Medicine (UCEM), University of Alabama at Birmingham, Birmingham, AL, United States

**Keywords:** swearing, physical performance, ergogenic, habituation, dosage

## Abstract

Swearing, or using taboo language with the potential to offend, has been shown to improve physical performance during short and intense tasks requiring strength and power development. While consistent ergogenic effects of swearing have been observed across studies, the mechanisms by which swearing impacts physical performance are not fully clear. Swearing has been shown to modulate physiological (i.e., heart rate, blood pressure, skin conductance), psychological (i.e., state disinhibition), and nociceptive (i.e., pain threshold, pain tolerance, pain perception) responses, thus making it plausible that these mechanisms allow swearing to positively impact physical performance. A variety of dosages of swearing (i.e., word used, intensity, frequency, quantity) have been reported to improve physical performance. Although habituation to the positive physical performance effects of swearing has not been explored formally through empirical research, habituation to swearing has been observed in other contexts. From a practical application standpoint, swearing represents a low-risk, effective, and inexpensive intervention that has the potential to acutely improve physical performance although the taboo nature of swearing may limit its utility in real-world situations. The purpose of the following review is to provide an overview of available evidence on swearing and physical performance and discuss likely underlying mechanisms. Exploration of different swearing approaches and habituation will also be highlighted and suggestions for future research will be discussed, to more comprehensively understand if swearing can be strategically used for performance enhancement.

## 1 Introduction

Swearing, or the use of potentially offensive taboo words (Beers Fägersten, 2012), represents a complex social and linguistic phenomenon that has existed for centuries. The degree of offensiveness of particular words has manifested as cultural constructs that have been largely dictated by societal and religious norms throughout history (Mohr, 2013). Due to the pressure to conform to societal norms, most individuals inhibit the use of swear words, leading to unique physiological and psychological consequences when swearing is used in specific contexts (Stapleton et al., 2022). Swearing has been found to elicit positive physiological, psychological, and social effects not achievable through conventional language (Stapleton et al., 2022). The positive outcomes associated with swearing include increased pain tolerance (Robertson et al., 2017; Stephens et al., 2009; Stephens and Robertson, 2020; Stephens and Umland, 2011), heightened humor (Beers Fägersten, 2012), enhanced credibility (Rassin and van der Heijden, 2005), strengthened social bonds (Beers Fägersten, 2012; Daly et al., 2004; Giffin, 2016; Stapleton, 2010), and improved memory (Jay et al., 2008; MacKay et al., 2004).

The evidence of using swearing as an ergogenic aid is relatively novel and limited, but the mechanistic study of swearing in other contexts bolsters the notion of possible performance-enhancing effects (Jiannine and Antonio, 2023; Stephens et al., 2018, 2022). Since the distinct impact swearing has on physical performance has only recently become a prominent topic of investigation, many questions remain unanswered regarding strategic use and real-world implications. Thus, the overall purpose of this review is to summarize existing evidence on how swearing impacts physical performance and draw insight from swearing studies in other contexts to form a basis for preliminary recommendations for swearing use while drawing attention to the need for future research.

## 2 Current view of swearing and physical performance

The initial investigation into the impact of swearing on physical performance, to our knowledge, occurred in 2018, where Stephens et al. (2018) conducted experiments examining how swearing affects strength and power performance. Participants in these studies chose their swear word by being asked for a swear word they might use in response to banging their head accidentally, then repeated their swear word every 3 s during a 30-s Wingate Anaerobic Power Test (Bar-Or, 1987). Participants also repeated their swear word for 10 s prior to testing their grip strength then continue to repeat their swear word during the grip strength test. These experiments found that swearing increased peak and average power on the Wingate Anaerobic Power Test by an average of 4.5% and improved grip strength by an average of 8% compared to repeating a non-swear word.

Subsequent studies have replicated these findings. Stephens et al. (2022) conducted a replication study on the effects of swearing on grip strength. Participants swore for 10 s prior to testing their grip and, again, swearing was found to improve grip strength by an average of 8% (+ 2.49 kg). Another experiment examined the effects of swearing on a different physical task, a chair push-up task, which is a body weight exercise requiring participants to raise their bodies and support their body weight on their hands and arms against the chair seat for as long as possible (Stephens et al., 2022). Participants were able to hold the chair push-up position for 10% longer when swearing, compared to repeating a neutral word. Jiannine and Antonio (2023) investigated the effects of swearing on grip strength, push-ups to fatigue, and wall sit exercise and plank exercise time to exhaustion. Participants were asked for a swear word that they would use in response to accidentally stubbing their toe. The majority of the participant chose “fuck” as their desired swear word. During each physical performance task, participants repeated their chosen swear word every 5 s throughout the task. Swearing resulted in a significant ergogenic effect, where the swearing improved grip strength by 9%, wall sit time to exhaustion by 22%, push-ups to fatigue by 15%, and plank exercise time to exhaustion by 12%. Collectively, these studies provide compelling evidence supporting the ergogenic impact of swearing on relatively short, intense physical tasks (Table 1). These findings suggest swearing could have meaningful impacts in real-world settings. For example, elite weightlifters typically increase their lower extremity strength by only 3.5% over the course of a year (Häkkinen et al., 1987), and the finishing times between the 1st and 8th place in the men's 100-meter sprint at the 2024 Summer Olympics differed by just 1.23% (Athletics at the 2024 Summer Olympics—Men's 100 Metres, 2024). These examples highlight the potential significance of the performance improvements observed with swearing in various physical contexts.

**Table 1:** Reviewed studies on how swearing influences physical performance.

**References**	**Conditions**	**Dosage/approach**	**Physical task**	**Primary findings**
Stephens et al. (2018)	Swear word, neutral word	Swear every 3 s during task	Wingate Anaerobic Power Test	4.5% ⇑ power
Stephens et al. (2018)	Swear word, neutral word	Swear for 10 s prior to task and during task	Grip strength	8% ⇑ grip strength
Stephens et al. (2022)	Swear word, neutral word	Swear for 10 s prior to task	Grip strength	8% ⇑ grip strength
Stephens et al. (2022)	Swear word, neutral word	Swear every 2 s prior to task	Chair push-up to hold exhaustion	10% ⇑ hold time
Jiannine and Antonio (2023)	Swear word, neutral word	Swear every 5 s during task	Grip strength; Wall sit to exhaustion; Push-ups to fatigue; Plank to exhaustion	9% ⇑ grip strength; 22% ⇑ wall sit time; 15% ⇑ push-ups; 12% ⇑ plank time

Conditions, specific dosages and approaches, physical task, and primary findings from each investigation are presented. ⇑ indicated an increase.

## 3 Potential ergogenic mechanisms

### 3.1 Physiological

Physiological arousal refers to the activation of bodily systems in response to stimuli, primarily through the autonomic nervous system, specifically the sympathetic branch. This heightened state is characterized by increased heart rate, blood pressure, respiratory rate, pupillary dilation, and skin conductance, collectively known as the “fight or flight” response. While the mechanisms by which swearing influences physical performance are not yet fully understood, research suggests that swearing can alter physiological mediators of exercise performance, by modulating the sympathetic nervous system. For example, Harris et al. reported that skin conductance responses, a marker of sympathetic activation, were higher when participants repeated a taboo word vs. a neutral word (Harris et al., 2003). Additionally, swearing has been shown to elicit greater speech-evoked pupillary responses than neutral word, further indicating heightened sympathetic arousal (Reilly et al., 2020). However, the precise role of swearing in sympathetic modulation remains unclear as other investigations have reported little to no change in markers of autonomic markers when swearing (Stephens et al., 2018).

Despite swearing being linked to sympathetic activation, only one study, to our knowledge, has successfully examined the ergogenic effects of swearing while monitoring outcomes reflective of autonomic changes during physical activity. Stephens et al. (2018) conducted dual experiments in the context of using swearing during anaerobic and strength exercises. The first experiment examined the effects of swearing on performance during a Wingate Anaerobic Test (WAnT) which is a maximal high-intensity cycle sprint test. Mean and peak power output during the WAnT increased in the swearing vs. non-swearing condition. Despite the improved physical performance that resulted from swearing, autonomic variables remained largely unchanged. That is, heart rate and blood pressure responses following the WAnTs remained similar (Stephens et al., 2018). At face value, this would suggest that sympathetic activity was unaffected by swearing during exercise. However, it is worth mentioning that observations of autonomic changes may have been negated by multiple factors. First, swear words were repeated throughout the exercise bout. The act of repetitively speaking results in an alteration in breathing behavior which invariably alters parasympathetic activity to the heart (Beer, 2023). Since words were repeated in both conditions, it is plausible that participants repeated words at similar rates which would have resulted in similar autonomic balance to the heart during the activity. Also, WAnTs are maximal in nature and typically result in pronounced stress responses. Participants in the experiment reach ~90–95% of their age-predicted maximum heart rate (Stephens et al., 2018). It is possible that the overwhelming amount of sympathetic activation initiated during the exercise “washed out” any differences in changes in heart rate and blood pressure measures possibly initiated by swearing. Thus, it appears that while swearing improves high-intensity exercise performance, the physiological mechanisms for enhanced performance may be independent or less reliant on sympathetic activation during exercise. In Stephens, Spierer and Katehis (2018) second experiment, participants completed maximal isometric handgrip tests while repeating their chosen swear word or a neutral word. Results showed that maximal isometric force increased. However, measures of autonomic activation were, again, unchanged. It is plausible that the autonomic measures utilized in these experiments were insufficiently sensitive to capture subtle changes in sympathetic activation, or it may be that the mechanisms by which swearing impacts physical performance is something other than sympathetic activation. However, it is clear that more systematic investigations on the physiological effects of swearing during physical effort are warranted.

### 3.2 Psychological

Psychological arousal refers to the activation of emotional and mental states, such as confidence, excitement, humor, or distraction. This type of arousal is closely linked to brain activity, particularly in areas like the amygdala, which plays a key role in processing emotions. Psychological arousal is often associated with physiological arousal, as the two can influence one another (Raz and Lahad, 2022). However, for the purpose of this mini-review, discussions of physiological and psychological mechanisms will be separated due to their distinct measurement methods. Physiological arousal is typically measured objectively, through metrics like heart rate, while psychological arousal is measured subjectively, such as through self-reports of confidence or emotional states.

Swearing induces a number of psychological changes that may impact physical performance. First, swearing has been shown to be associated with emotional arousal (Janschewitz, 2008). This association between emotional arousal and swearing is likely dependent on societal norms and degree of taboo nature (Janschewitz, 2008). Stephens and Robertson (2020) showed that swearing induced higher emotional and humor ratings compared to neutral words. While mechanisms for these responses are still unclear, it has been established that emotional arousal is primarily reflective of amygdala activity (Hamann and Mao, 2002). Interestingly, responses to verbal stimuli appear to be localized to the left amygdala region which is in support of previous findings suggesting the left region is associated with verbal processing and sustaining emotional perceptions (Baas et al., 2004). Similarly, effort and motivation appear to be tightly linked to amygdala activation which may mediate physical performance (Smith and Torregrossa, 2021). Indeed, auditory and verbal stimuli (e.g. music) that induce heightened arousal and motivation have been well-established to improve physical performance (Ballmann, 2021; Ballmann et al., 2021). Thus, swearing may invoke emotional responses that increase amygdala-mediated arousal resulting in the amplification of motivation and physical effort.

Another psychological mechanism for the observed effect of swearing on physical performance may be due to an increase in state disinhibition, a state in which someone is less likely to hold back. This has been similarly suggested in other non-taboo verbiage during physical effort. O'Connell et al. (2014) showed that verbal grunts helped tennis players hit the ball with greater power (mean increase 19%-26%) and by Welch and Tschampl (2012) in their study of hand grip strength accompanied by shouting (mean increase 7%). Research by Stephens et al. (2022) examined the possibility that psychological mechanisms produced the ergogenic effects of swearing. It was discovered that swearing increases state disinhibition via a rise in risky behavior, also likely under the control of amygdala activation. This, in turn, increases psychological flow, positive emotion, distraction, and self-confidence. However, contrary to Stephens et al. (2022) hypothesis, a mediation analysis revealed no evidence that risky behavior mediated the positive effects of swearing on physical performance. While swearing does appear to elevate risky behavior, it does not appear to be the mechanism through which swearing enhances physical performance. Further research is needed to investigate the potential that psychological arousal mediates the ergogenic effects of swearing.

### 3.3 Nociception

**Figure 1:**
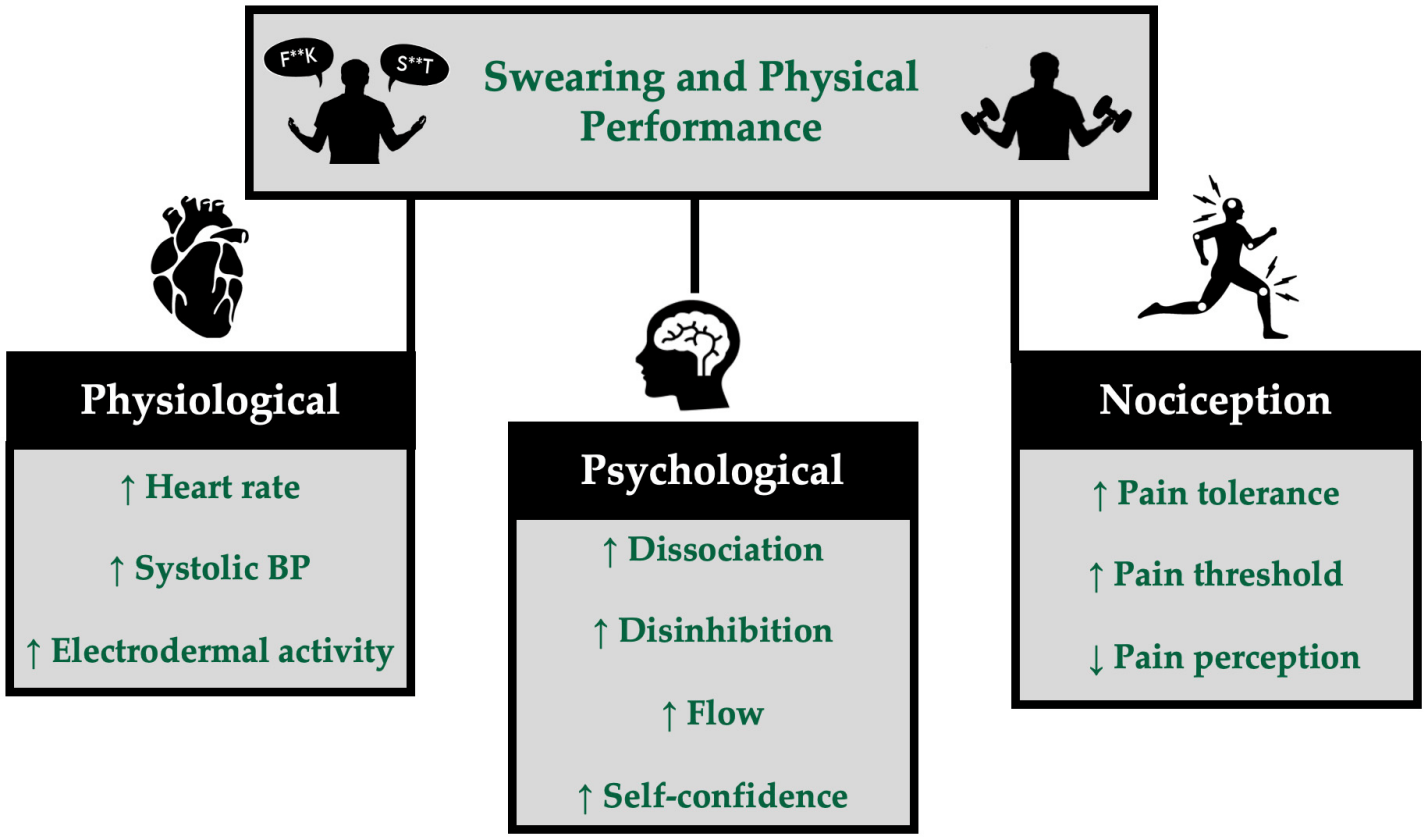
Plausible mechanisms by which swearing may impact physical performance and responses to exercise. Physiological, psychological, and nociceptive alterations underpin the ergogenic effects of swearing and can be distinct or synergistic in action.

Another possible mechanism by which swearing improves physical performance includes swearing-induced hypoalgesia. Swearing-induced hypoalgesia is an effect that has been observed across studies (Robertson et al., 2017; Stephens et al., 2009; Stephens and Robertson, 2020; Stephens and Umland, 2011; Hostetter and Rascon-Powell, 2022; Philipp and Lombardo, 2017). The findings of these studies suggest that swearing increases pain threshold (Stephens and Robertson, 2020; Hostetter and Rascon-Powell, 2022), increases pain tolerance (Robertson et al., 2017; Stephens et al., 2009; Stephens and Robertson, 2020; Hostetter and Rascon-Powell, 2022), and decreases the perception of pain (Stephens and Robertson, 2020; Hostetter and Rascon-Powell, 2022). Only one study, to our knowledge, examined the effect of swearing on physical performance while also assessing pain perception. Even when swearing was found to increase grip strength by Stephens et al. (2018), a decrease in pain perception was not observed. However, swearing-induced hypoalgesia may still be a possible mechanism. Although pain perception ratings were similar when repeating a swear word or a non-swear word while testing grip strength, it may be that the swearing rendered the pain and discomfort of the grip strength task more tolerable such that a greater amount of force could be exerted while the pain rating remained stable. Physical performance tests require a person to attempt maximal effort, which can be uncomfortable and sometimes even painful. It is possible that reduced pain perception due to swearing underlies the ergogenic effect by making it more tolerable to complete a strenuous task. It is also plausible that the mechanism behind swearing's ergogenic effect include a combination of sympathetic activation, hypoalgesia, and increased state disinhibition (Figure 1). The exact pathways remain unclear, necessitating further investigation.

## 4 Swearing dosage

Dosage appears to be an important factor for interventions aimed at improving physical performance. Dosages of an intervention, include, but are not limited to, the intensity, frequency, and quantity of the intervention. For example, when considering resistance training to improve physical performance, quantity dosage appears to be more important than frequency dosage of resistance training for improvements in strength and hypertrophy (Grgic et al., 2018; Schoenfeld et al., 2019).

The studies examining the impact of swearing on physical performance have allowed participants to self-select their swear word by asking participants to choose a swear word they would use in response to “banging their head accidentally” (Stephens et al., 2018) or “accidentally stubbing their toe” (Jiannine and Antonio, 2023). “Fuck” and “shit” are the most commonly selected swear words, with one study reporting 51.5% of participants selecting “fuck” and 38% selecting “shit” (Jiannine and Antonio, 2023). With respect to speech volume, experiments consistently instruct participants to use a clear voice with normal volume, and not to whisper or shout (Jiannine and Antonio, 2023; Stephens et al., 2018, 2022). With respect to frequency dosage, studies have asked participants to swear every 2 s (Stephens et al., 2022), every 3 s (Stephens et al., 2018), every 5 s (Jiannine and Antonio, 2023), or “at a steady pace” (Stephens et al., 2018). Additionally, participants in these studies were instructed to swear just prior to performing the physical task, throughout the physical task, or swear both prior to and throughout the physical task. This variety of swearing frequencies has led to different swearing quantities across experiments. The quantity of swearing in these study have ranged from 2 to 3 total swear words to 45 swear words (Jiannine and Antonio, 2023; Stephens et al., 2018, 2022).

The swearing dosages have varied across these experiments; however, these studies consistently found improved physical performance with swearing. This suggests that a variety of swearing dosages positively impact physical performance (Table 1).

### 4.1 Interference from habituation

Habituation, described as the progressive decrease in a response to a repeated stimulus (Rankin et al., 2009), is a phenomenon that has not yet been explored in the context of swearing and its ergogenic effects. However, habituation has been studies in other contexts. Stephens and Umland (2011) revealed habituation to swearing's hypoalgesic effects, with those that swear more frequently in their daily lives experiencing a lesser hypoalgesic response when they swear. Philipp and Lombardo (2017) found that swearing for 2 min reduced feelings of pain; however, those that swore less often in their daily lives experienced a greater hypoalgesic effect compared to those who swear more often. This implies that overuse of swearing in everyday situations lessens its effectiveness as a short-term intervention to reduce the perception of pain.

Habituation to swearing in various contexts has been observed in other studies. Lafreniere et al. (2022) examined the impact of swear words in online reviews within Yelp and Amazon platforms. It was found that reviews with swear words were perceived as more useful than reviews without swear words, but a habituation effect was also observed. The presence of a few swear words in online reviews increased the usefulness of those reviews, while many swear words did not. This suggests that readers may become tolerant to swearing when reviews have too many swear words. In another study, MacKay et al. (2004) used the taboo Stroop test to determine if habituation to swear words occurs when participants are shown swear words with increased repetition. The taboo Stroop test requires participants to name the color of randomly intermixed swear and non-swear words appearing on a screen. There was a strong taboo Stroop effect, that is, participants demonstrated slower reaction times when naming the colors of swear words compared to neutral words. However, there was a reduced Stroop effect with increased repetition of specific swear words across the trials. MacKay et al. (2004) suggest that emotional arousal contributes to the Stroop effect, and repeated exposure to a swear word will habituate the emotional reaction and, therefore, reduce the Stroop effect.

## 5 Conclusions

Swearing has been shown to improve physical performance in tasks that are relatively short and intense, and this effect has been repeated across experiments, suggesting that it may be a reliable effect. However, all available studies have been conducted in laboratory settings and in controlled environments. It is unknown whether the performance improvements that are associated with swearing occur in more naturalistic real-world settings; outside of a laboratory.

Future research should explore the impact of swearing on diverse physical tasks, variations of dosage parameters, and potential habituation patterns. Physical tasks other than short and intense tasks, including aerobic endurance, balance and other skill-base tasks, and tasks involving manual dexterity, need to be examined to better comprehend the ergogenic effects of swearing. To better apply swearing in real-world scenarios, studies should examine variations in dosage, by modulating variables such as intensity (i.e., Repeating “fuck” vs. “crap”), mode (ie. Spoken vs. heard vs. read swear words), and frequency (i.e., Swearing every 2 s vs. every 60 s). Research should explore habituation patterns of swearing over specific timeframes, such as across several minutes, days, and weeks. Determining whether someone can be re-sensitized to swearing after being habituated to swearing would prove beneficial in the real-world application of these ergogenic effects. Mechanisms research holds promise in discovering the intricacies of swearing's effects, potentially providing insights into optimizing ergogenic outcomes and preventing habituation. Studies that evaluate *how* swearing works, not just *if* swearing works are of considerable importance. Mechanisms research aims to improve swearing outcomes by tailoring swearing to the specific needs of each individual. This comprehensive research approach is essential for advancing our understanding of when, how, and if swearing can be strategically employed to enhance physical performance.

Swearing provides a means by which to improve physical performance in relatively short, intense physical tasks, such as testing grip strength and completing push-ups to fatigue. Swearing can be easily utilized, is cost-effective, and appears to be a potent ergogenic intervention. While the evidence described in this review supports swearing for improved physical performance, this may not be feasible for all individuals or in all situations. The taboo nature of swearing may limit its utility in real-world situations. Many fitness centers and competition settings prohibit offensive behavior, including swearing. Thus, individuals should consider the context in which swearing is utilized when considering employing this ergogenic method. The simple modification of swearing internally or quietly enough where others are unable to hear, may ultimately lead to a greater utility of swearing for enhanced performance in real-world setting; however, this modification has not been explored by empirical research and there is no data to support swearing quietly is effective. While the mechanisms for physical performance improvements from swearing are not fully understood, it appears to be a combination of sympathetic activation, hypoalgesia, and increased state disinhibition. Further research is essential for advancing our understanding of when, how, and if swearing can be strategically employed to enhance physical performance.
